# Regulation and therapy, the role of JAK2/STAT3 signaling pathway in OA: a systematic review

**DOI:** 10.1186/s12964-023-01094-4

**Published:** 2023-04-03

**Authors:** Bo Chen, Ke Ning, Ming-li Sun, Xin-an Zhang

**Affiliations:** College of Exercise and Health, Shenyang Sport University, Shenyang, China

**Keywords:** Osteoarthritis, JAK2/STAT3, Pathway, Mechanism, Targeted therapy

## Abstract

Osteoarthritis (OA) is a multifactorial chronic disease primarily characterized by the degeneration of articular cartilage. Currently, there is a lack of effective treatments for OA other than surgery. The exploration of the mechanisms of occurrence is important in exploring other new and effective treatments for OA. The current evidence shows that the Janus kinase 2 (JAK2)/signal transducer and activator of transcription 3 (STAT3) signaling pathway plays a vital role in cytogenesis and is involved in OA progression. The terms “JAK2”, “STAT3”, and “Osteoarthritis”were used in a comprehensive literature search in PubMed to further investigate the relationship between the JAK2/STAT3 signaling pathway and OA. This review focuses on the role and mechanism of JAK2/STAT3 signaling in cartilage degradation, subchondral bone dysfunction, and synovial inflammation. In addition, this review summarizes recent evidence of therapeutic approaches to treat OA by targeting the JAK2/STAT3 pathway to accelerate the translation of evidence into the progression of strategies for OA treatment.

**Video abstract**

**Supplementary Information:**

The online version contains supplementary material available at 10.1186/s12964-023-01094-4.

## Introduction

**Fig. 1:**
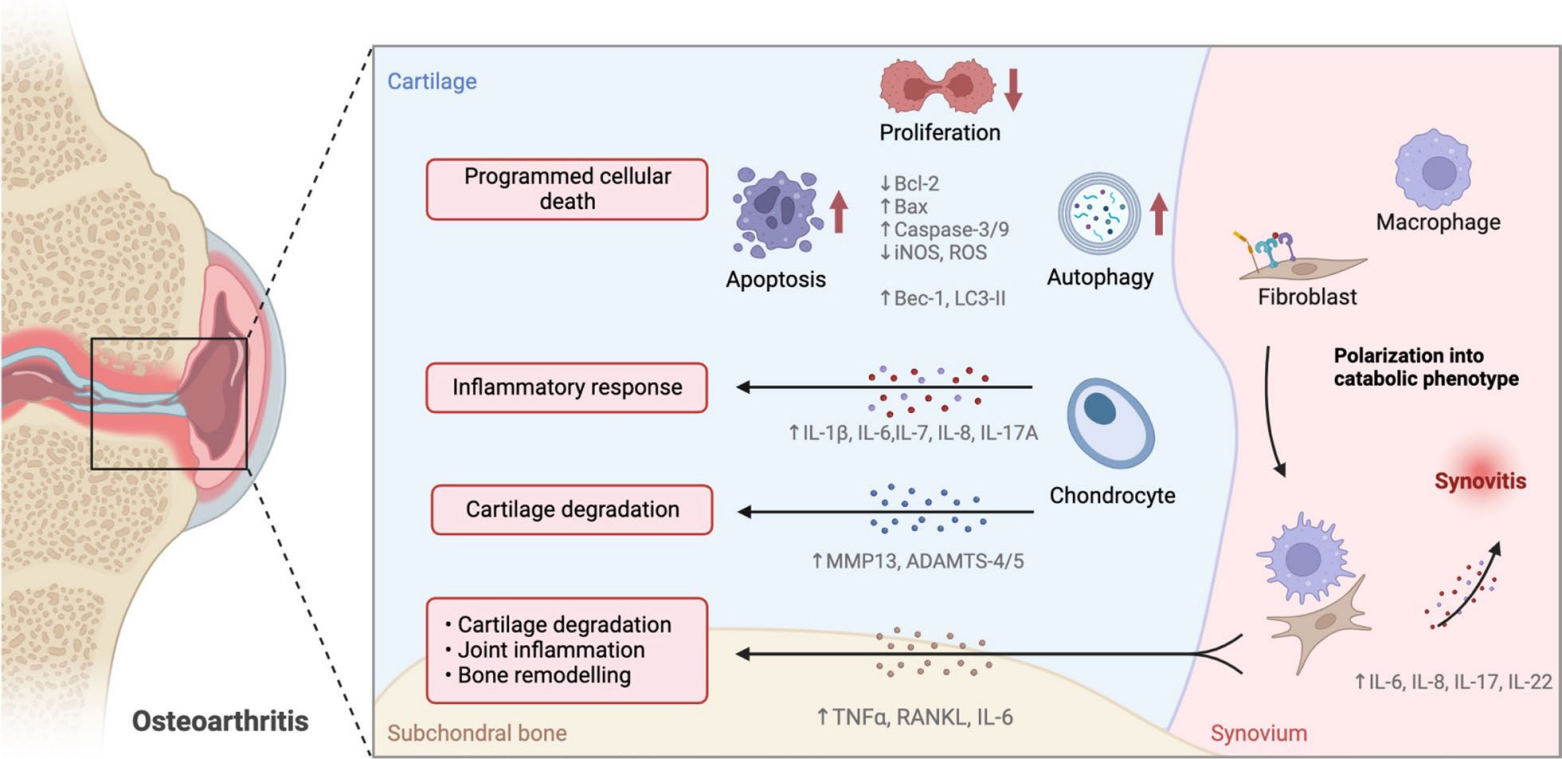
The pathology of osteoarthritis. The pathology of OA is typically characterized by articular cartilage degeneration, subchondral bone sclerosis and synovial lesions. Cartilage degradation is caused by programmed cellular death (apoptosis, proliferation, and autophagy) or inflammatory response. ↑: up-regulation;↓: down-regulation (Created with BioRender.com.)

Osteoarthritis (OA) is a degenerative joint disease occurring in the elderly population. Its pathology is typically characterized by articular cartilage degeneration, subchondral bone sclerosis and synovial lesions [1] (Fig. 1). The main symptoms of OA are edema, chronic pain and limited joint movement [1–3]. Recently, some evidence have indicated that globally approximately 240 million people have symptomatic OA, which is an important cause of physical disability and poor quality of life [4, 5]. Particularly, patients with advanced OA have to choose surgical treatment and thus face a heavy financial burden [6]. In the early stages of OA, some non-surgical treatments, including physical therapies, nonsteroidal anti-inflammatory drugs (NSAIDs) and glucosamine, are used to treat patients with OA, but do not reverse the progression of this disease [6]. This is due to the nature of OA as a polygenic disease and its unclear molecular mechanisms. Multiple signaling networks have been reported to be involved in the pathogenesis of OA [7–9]. Therefore, a comprehensive understanding of the molecular networks that regulate OA pathogenesis is important for the progression of more effective OA therapies.

**Fig. 2:**
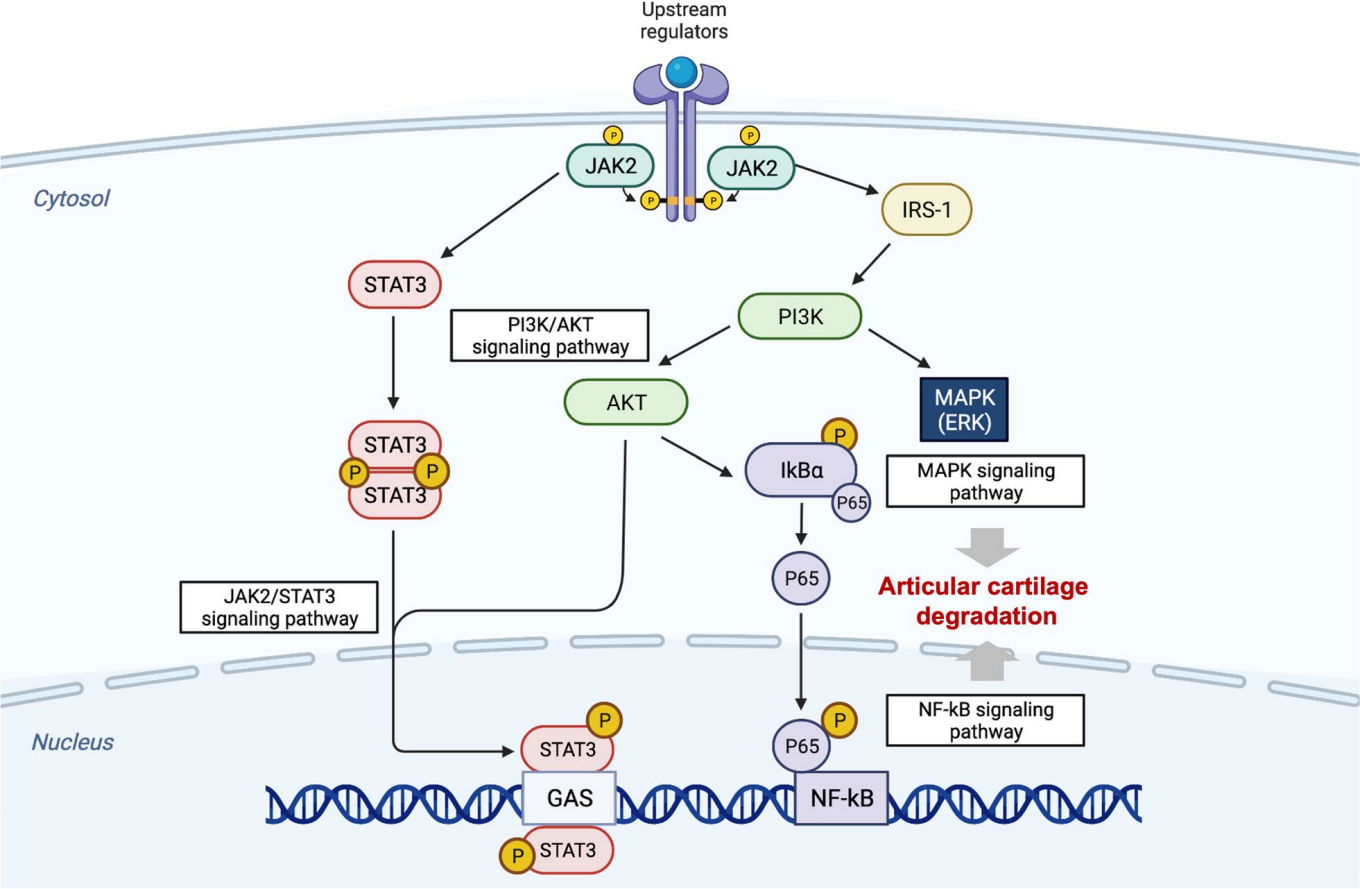
The JAK2/STAT3 signaling pathway interplayed with other signaling pathways in chondrocytes. Phosphorylated STAT3, activated by JAK2, is translocated to the nucleus, and interacting with NF-kB, AKt/PI3K, and MAPK signaling pathways, which are involved in articular cartilage degradation. (Created with BioRender.com.)

The JAK2/STAT3 is involved in the initiation and progression of inflammatory responses and immune responses in diverse pathological processes and plays an important role in multiple diseases [10]. Janus kinase 2 (JAK2) belongs to the JAK family and is multidirectional and associated with physiological processes [11]. JAK2 is expressed in a variety of tissues and cells and is involved in cell differentiation, apoptosis, and immune regulation, with many essential roles. Signal transducer and activator of transcription 3 (STAT3), a member of the STAT family [12], acts as a transcription factor by binding DNA under a variety of pathological conditions [13]. Recently, a study described that STAT3 expression is associated with the progression of several bone-related diseases, including osteoporosis, osteoarthritis and bone progression and repair [14]. Mechanistically, in the cytoplasm, JAK2 acts as an anchor site for STAT3. Inactive JAK undergoes conformational changes and is converted to active JAK (p-JAK), which in turn phosphorylates JAK residue receptors in p-JAK in the cytoplasm, leading to the recruitment of STAT3 binding sites [12]. STAT3 is translocated from the cytoplasm to the nucleus as a dimer and plays a key role in the transcription of target genes, influencing downstream transcription and protein production [11, 15]. The JAK2/STAT3 pathway is a well-conserved pathway that is closely related to the expression of genes, including cell growth, survival and apoptosis. In addition, the JAK2/STAT3 signaling pathway is relevant to the initiation and progression of the disease, such as cancer neurological, and immune-inflammatory conditions [16–25]. Several studies have shown that the pathogenesis of OA is, at least in part, the result of interactions between JAK2/STAT3 and multiple signaling pathways. Based on recent literature, signaling pathways including JAK2/STAT3, NF-kB, PI3K/AKT and MAPK exhibit abnormal activity and interactions with each other that is an important participant in OA progression (Fig. 2). This indicates that JAK2/STAT3 pathway may be a prospective target for the therapy of OA.

In this review, 32 core articles were collected from PubMed, Web of Science, Embase and other platforms with "JAK2 OR STAT3" and “osteoarthritis” as keywords in recent years.. The main selection criterion was the application of regulatory factors for the treatment of OA by regulating cartilage, subchondral bone, and synovium via the JAK2/STAT3 signaling pathway. The review systematically presents the regulatory role of the JAK2/STAT3 signaling pathway in three aspects of OA, including cartilage, subchondral bone and synovium, and therapeutic prospects of targeting the JAK2/STAT3 signaling pathway in OA. The purpose of this study is to provide a further reference for novel perspectives on the treatment of OA and the clinical application of targeted drugs related to the JAK2/STAT3 signaling pathway.

## Effect of the JAK2/STAT3 signaling pathway on OA

**Fig. 3:**
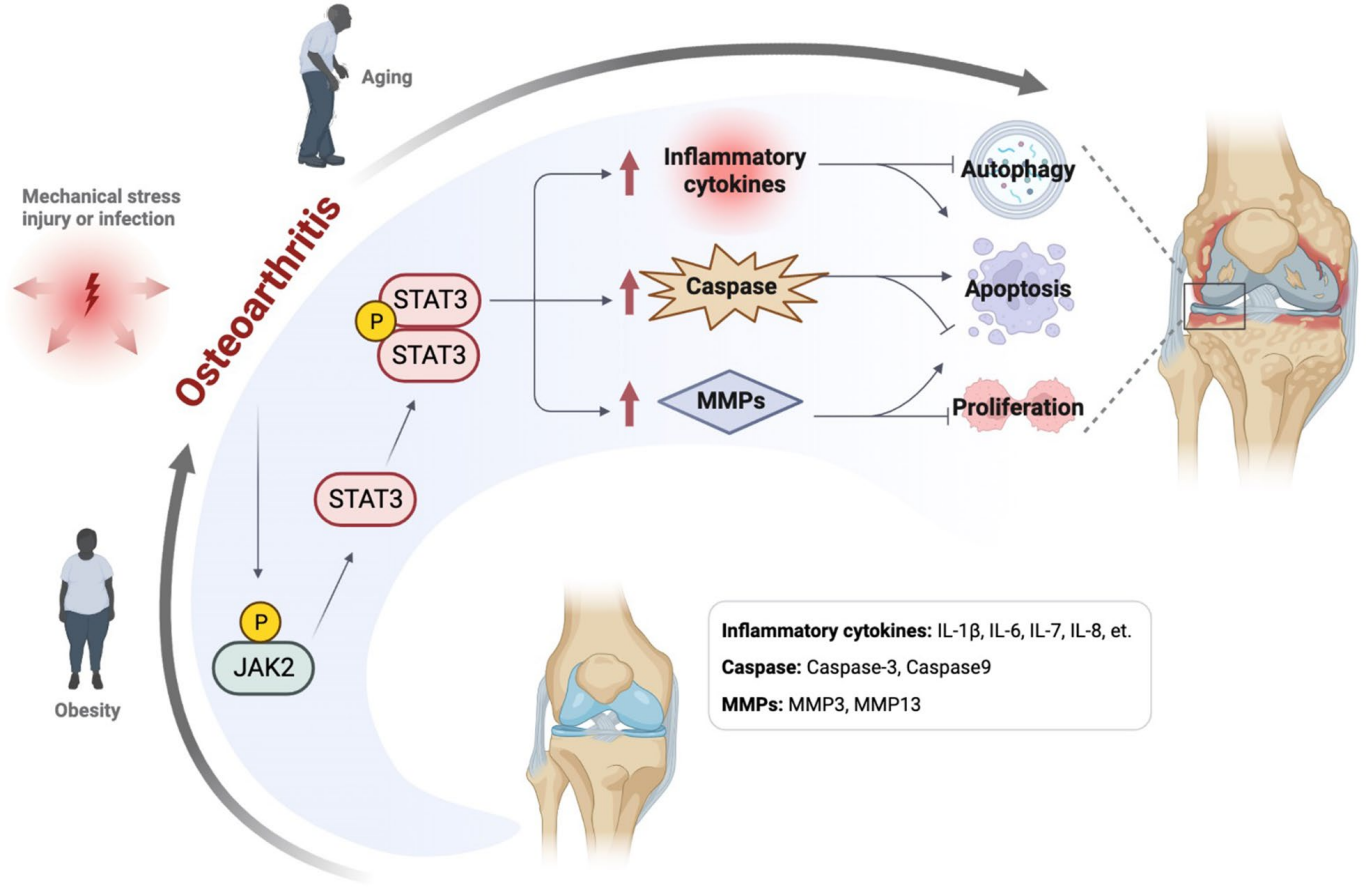
Relationship between JAK2/STAT3 signaling pathway and Osteoarthritis. Obesity, mechanical stress, and aging are risk factors for OA. The JAK2/STAT3 signaling pathway modifies the pathological process of OA by regulating related cytokines. ↑: up-regulation (Created with BioRender.com.)

Many risk factors contribute to OA, such as obesity, aging and joint injuries. The relationship between OA and JAK2/STAT3 signaling pathway is closely related (Fig. 3) (Table 1). The specific mechanisms involved are described in the following subsections.

**Table 1:** The role of the JAK2/STAT3 signaling pathway in OA

Study type	Upstream regulator	Target cell/tissue	Effect on JAK2/STAT3	Mechanism	Consequence	References
In vitro study	HIF-1α	MLO-Y4	Activation	RANKL↑	Promoted osteocyte-mediated osteoclastic differentiation in vitro	[53]
In vitro and vivo study	DA	C28/I2 cells DMM-induced OA C57BL/6 mice	Inhibition	iNOS↓, COX-2↓, MMP-1↓, MMP-3↓, MMP-13↓ COL-II↑, AGG↑	Delayed articular cartilage degradation in OA Abrogated the degradation of cartilage matrix and reduced Osteoarthritis Research Society International scores	[34]
In vitro and vivo study	TRIM59	Primary human OA chondrocyte ACLT-induced OA SD rats	Inhibition	IL-1β↓, MMP-13↓, COL-II↑, AGG↑	Maintained the balance of ECM and inhibited the production of proinflammatory cytokine and apoptosis. Enhanced cell viability. Alleviated IL-1β-driven cartilage matrix degradation	[35]
In vitro and vivo study	INSR	Mice chondrocytes ACLT-induced OA C57BL/6 J mice	Inhibition	COL-II↑, GAG↑, p-JAK2↑, p-STAT3↑ ADAMTs-4↓, MMP-3↓, MMP-13↓	Decreased the chondrocytes apoptosis and ameliorated the pathological symptoms of OA	[36]
In vitro study	Inc RNA DANCR	Human chondrocytes	Inhibition	IL-6↓, IL-8↓	Promoted inflammation, cell proliferation, and suppressed apoptosis	[41]
In vitro and vivo study	DUSP19	Rat chondrocytes ACLT-induced OA SD rats	Inhibition	p-JAK2/JAK↓, p-STAT3/STAT3↓, Bax↑, Caspase-3↑, Bcl-2↓, MMP-3↓, MMP-9↓, MMP-13↓	Inhibited chondrocytes apoptosis and MMPs expression	[45]
In vivo study	PTH (1–34)	Collagenase-induced OA C57BL/6 mice	Inhibition	COL-II↑,AGG↑,ADAMTS-4↓,MMP-13↓,Caspase-3↓,P53,Bax↓, P-JAK2, P-STAT3↓	Ameliorated cartilage degeneration and subchondral bone deterioration	[31, 32]
In vitro and vivo study	Leptin	Rat chondrocytes C28/I2 and T/C-28a2 cells Human FLS ACLT-induced OA SD rats	Inhibition	MMP-9↑, MMP-13↑, ROS↑, iNOS↑, Bax↑, Bcl-2↓, Belin-1↓, LC3↓	Induced apoptosis. Regulated expression of Frizzled receptors in chondrocytes. Promoted expression of IL-6 and IL-8	[42, 48, 63, 79, 83, 84]
In vitro and vivo study	BCP	Mice chondrocytes MNX-induced OA C57BL/6 mice Human cartilage explants	Inhibition	IL-6↓, MMP-13, MMP-3, ADAMTS-4, ADAMTS-5	BCP crystals and IL-6 form a positive feedback loop leading to OA	[40]
In vitro study	CXCL16	Human FLS	Activation	RANKL↑	Upregulated RANKL expression	[64]
In vitro study	Ghrelin	Human chondrocytes	Inhibition	MMP-3↓, ADAMTs-4↓, ADAMTS-5↓, COL-II↑, AGG↑	Protected articular cartilage matrix destruction	[30]
In vitro study	AGEs	Porcine chondrocyte	inhibition	MMP-3↓, ADAMTs-4↓, ADAMTS-5↓, COL-II↑, AGG↑	Prevented AGE-mediated decrease of COL-II and AGG	[33]
In vitro study	IL-22/IL-22R1	Human FLS	Inhibition	inhibited JAK2/STAT3 p-STAT3↑, MMP-1↑, S1001A8/A9↑	Amplified FLS activation	[61]

### JAK2/STAT3 signaling pathway and cartilage in OA

#### Cartilage homeostasis

Cartilage homeostasis is a state of equilibrium in the synthesis of extracellular matrix (ECM) that is critical to overall joint health [5]. The progression of cartilage homeostasis is characterized by the upregulation of collagen-II (COL-II) and aggrecan (AGG) levels, along with a decrease in a disintegrin and metalloprotease with thrombospondin motifs (ADAMTs) and matrix metalloproteinases (MMPs) [26]. MMPs family(MMP1, MMP-3, MMP-9, MMP13) and ADAMTs family(ADAMTs-4, ADAMTs-5) cause matrix degradation and disrupt cartilage homeostasis [27]. Previous studies exposed that the expression of the JAK2/STAT3 pathway was abnormally activated in osteoarthritic cartilage relative to normal cartilage tissue. Lu et al. constructed an OA model using human articular chondrocyte C28/I2 cells and human primary chondrocytes under IL-1β stimulation. They found that inhibition of JAK2 influenced the expression of its downstream molecule STAT3, which exhibited that significant reduction in AGG loss and loss of chondrocyte cellularity. This suggests that the JAK2/STAT3 signaling pathway is involved in the imbalance of OA cartilage homeostasis [28]. Rong et al. inserted shJAK2 into OA chondrocytes by transfection and confirmed the imbalance of OA cartilage has been reversed. They showed that knockdown of JAK2 eliminated the negative impacts of chondrocytes migration and apoptosis and promoted proliferation of chondrocytes [29]. The above experiments further demonstrated the important role of JAK2 in OA cartilage. Liu et al. found that the addition of a JAK2-specific inhibitor eliminated IL-1β-induced phosphorylation of STAT3 in primary chondrocytes [30]. Inhibition of the JAK2/STAT3 pathway prevented an increase in the expression of MMPs and further reversed the imbalance in cartilage homeostasis [30]. In another study, Shao et al. detected the expression of JAK2 and STAT3 in different groups of cartilage weight-bearing areas by immunohistochemistry and found that the expression levels of JAK2 and STAT3 in OA cartilage tissue were significantly higher than those in normal cartilage. It was further confirmed that high expression of JAK2 and STAT3 decreased COL-II levels and caused cartilage matrix damage [31, 32]. Besides, Huang et al. assessed the gene expression of MMPs and ADAMTs by RT-PCR and applied the histochemistry or immunoblotting analysis to determine the expression of STAT3, COL-II, AGG, and proteoglycan in the porcine cartilage fragments. They found that JAK2 inhibitors blocked the expression of MMP13, ADAMT4, and ADAMT5. Meanwhile, inhibition of JAK2 prevented the decrease of COL-II in chondrocytes and AGG degradation in cartilage fragments. Moreover, interference of STAT3 expression inhibited MMP13 and ADAMTS enzyme activities and mRNA levels [33]. Proteins such as COL-II, MMPs and AGG are typical markers of cartilage homeostasis. The above studies suggest that the JAK2/STAT3 pathway is closely associated with endochondral homeostasis in articular cartilage. In addition, the role of the JAK2/STAT3 pathway in maintaining endochondral homeostasis is regulated by a variety of factors. For example, an important neurotransmitter, dopamine (DA), could inhibit IL-1β-induced phosphorylation of the JAK2/STAT3 pathway and activation of NF-kB in vitro experiments, delaying cartilage degradation [34]. In another study, Teng et al. demonstrated that triple motif-containing 59 (TRIM59), a member of the triple motif-containing (TRIM) protein superfamily, blocked the phosphorylation of JAK2 and STAT3, thereby attenuating cartilage matrix degradation during OA progression [35]. In a collagenase-induced OA mouse model, Shao et al. found that parathyroid hormone (PTH) (1–34) could promote ECM anabolism through the downregulation of JAK2/STAT3 pathway. Further studies have shown that PTH (1–34) affected the transcription and translation of JAK2/STAT3 and did not affect its phosphorylation [31, 32]. There is another hormone, Ghrelin, reduced IL-1β-induced expression of MMP3, MMP13, ADAMTS-4 and ADAMTS-5 in a concentration-dependent manner and inhibited the degradation of COL-II and AGG. Further studies showed that the resistance of Ghrelin to the IL-1β-induced effects was achieved by inactivating the JAK2/STAT3 pathway [30]. Huang et al. [33] treated porcine chondrocytes and cartilage explants with advanced glycation end products (AGEs) for 24–48 h and found that AGEs could maintain ECM homeostasis by blocking the JAK2/STAT3 pathway, demonstrating that the JAK2/STAT3 pathway was essential for AGEs-mediated cartilage matrix damage. Additionally, insulin receptor (INSR) was mediated by Kruppel-like factor (KLF)-4 (KLF4) and DNA methylation maintenance of cartilage homeostasis via suppression of the JAK2/STAT3 signaling pathway [36]. Collectively, inhibition of JAK2/STAT3 could promote ECM anabolism and maintain cartilage homeostasis. Therefore, further study of the molecular mechanism of JAK2/STAT3 in cartilage homeostasis is necessary for the treatment of OA.

#### Inflammatory response

The inflammatory response usually occurs in conjunction with OA pathogenesis and OA-related symptoms. During the progression of OA, large amounts of inflammatory factors (IL-1β, IL-6, TNF-α, and IL-8) produced in chondrocytes or synovial cells can accelerate cartilage degradation [37]. Particularly, IL-1β can cause intense inflammatory responses by activating complex pathway networks [38]. Numerous studies identified that JAK2 and STAT3 were rapidly phosphorylated under the stimulation of IL-1β. Moreover, several biological and chemical compounds exerted both inhibitory effects on IL-1β-induced the phosphorylation of JAK2 and STAT3, and inflammatory responses. This suggested that the JAK2/STAT3 pathway might regulate the initiation of inflammatory responses. Teng et al. imposed IL-1β to treat primary human OA chondrocytes and observed that IL-1β significantly enhanced the level of inflammatory cytokines TNF-α and IL-6. They showed that TRIM59 reversed this situation by inhibiting the JAK2/STAT3 signaling pathway [35]. In addition to IL-1β, IL-6 also induces an inflammatory response in chondrocytes. Wang et al. conducted an OA model with IL-6-treated chondrocyte in vitro and found that Angiotensinogen (AGT) promoted IL-6-induced inflammatory responses of chondrocytes via activating the JAK2/STAT3 pathway. Inhibition of JAK/STAT could reverse the high level of IL-1β, and nitrite induced by IL-6 [39]. In another study, Sonia Nasi et al. found that basic calcium phosphate (BCP) crystals stimulated IL-6 secretion in chondrocytes, further amplified in an autocrine loop through activation of JAK2/STAT3 signaling pathway [40]. Zhang et al. found that inhibition of lncRNA DANCR reduced not only IL-6 but also IL-8 expression by the JAK2/STAT3/miR-216a-5p signaling pathway in cartilage samples from OA patients [41]. Tong et al. [42] followed the same view that the high expression of IL-8 in OA was concentration and time-dependent and was attenuated by JAK2 inhibitors or STAT3 siRNA. Besides, the inhibitors of JAK2 could reduce the expression of IL-6 and IL-7 in chondrocytes of OA C57BL/6 mice [31]. The above results indicated that JAK2/STAT3 is a critical pathway that modulates inflammatory responses in chondrocytes, the specific mechanisms of the JAK2/STAT3 pathway in OA should be further explored in in-depth studies.

#### Programmed cellular death

Aggregation of inflammatory responses may lead to programmed cell death, mainly including the aberrant levels of cell proliferation, apoptosis, and autophagy that exerted a disruptive influence on cartilage integrity and progression [43]. JAK2/STAT3 signaling pathway plays an important regulatory role in articular chondrocyte survival and apoptosis. Zhang et al. reported that the lncRNA DANCR was found to slow down the progression of OA by the suppressing JAK2/STAT3/miR-216a-5p pathway and promoting the proliferation and preventing chondrocytes apoptosis [41]. Agreeing with Zhang, Rong et al. [29] suggested that Hypo-sEV miR-216a-5p modulated chondrocyte proliferation, migration, and apoptosis inhibition via inactivating the JAK2/STAT3 pathway. In another in vitro experiment, CHON-001 chondrocytes were treated with IL-1β to mimic OA. Zhang et al. found that CIRC_PDE1C promoted C–C motif chemokine ligand 2 (CCL2) expression by competitively binding to miR-224, which activated the downstream JAK/STAT3 pathway and led to OA cartilage degradation [44]. Additionally, some hormones could exert similar anti-apoptosis effects as noncoding RNA via inhibiting the JAK2/STAT3 signaling pathway. An in vivo study by Shao et al. conducted experiments on chondrocytes in a mouse OA model and confirmed that PTH (1–34) could suppress chondrocyte apoptosis by downregulating the expression of caspase-3 via inhibiting the JAK2/STAT3 pathway. Moreover, they found that PTH (1–34) did not affect JAK2/STAT3 phosphorylation but instead affected JAK2/STAT3 transcription and translation [31]. Numerous studies have shown that IL-1β treatment could induce apoptosis of chondrocytes and trigger the activation of JAK2/STAT3 signaling pathway [31, 32]. Recently, multiple studies have reported that inhibition of the JAK2/STAT3 signaling pathway is able to rescue IL-1β-induced apoptosis and reduce OA cartilage degradation [35, 36, 45]. Therefore, the JAK2/STAT3 pathway positively mediated chondrocyte apoptosis. And the inhibition of JAK2/STAT3 pathway could protect against OA by reducing apoptosis and enhancing the proliferation of chondrocytes. Apart from regulating chondrocyte proliferation and apoptosis, JAK2/STAT3 could alleviate cartilage degradation by mediating autophagy. The transition process from autophagy to apoptosis impacts chondrocytes in the OA progression [46]. In contrast to apoptosis, autophagy dominates intracellular homeostasis and may have complex mechanisms at diverse stages of progression [47]. Zhang et al. [48] treated chondrocytes with JAK2 inhibition and observed that JAK2 inhibition increased the expression of autophagy markers Beclin-1 (Bec-1), autophagy protein 5 (ATG5), and microtubule-associated protein light chain 3 (LC-3), in the apoptosis of SD rat’s primary chondrocytes induced by leptin. Thus, programmed cellular death plays an important role in the pathogenesis of OA. The relationship between programmed cellular death and the JAK2/STAT3 signaling pathway in the progression of OA deserves further exploration.

### JAK2/STAT3 signaling pathway and subchondral bone in OA

Subchondral bone, the bone component under calcified articular cartilage, protects articular cartilage from external mechanical loads by distributing the loads evenly over the joint surface [49, 50]. A growing number of studies have noted that the progression of OA is regulated by subchondral bone–cartilage crosstalk [49, 50]. Microstructural alterations in subchondral bone are responsible for cartilage instability and lead to cartilage degeneration over time. The subchondral bone and osteochondral junction may be subjected to inappropriate external mechanical loading, thereby compromising their integrity [51]. Some studies have shown that the JAK2/STAT3 signaling pathway played an important role in subchondral bone remodeling. The phosphorylation of JAK2 and STAT3 could facilitate osteoblast differentiation [52]. The activity of osteoclast and osteoblast played an important regulatory role in bone modeling, reconstruction, and dynamic homeostasis. Zhu et al. conducted that the murine long bone-derived osteocytic Y4 cell line (MLO-Y4) cells stimulated by hypoxia stably expressed hypoxia-inducible factor-1α (HIF-1α). The results showed that HIF-1α enhanced the level of RANKL by activating JAK2/STAT3 pathway in MLO-Y4 and facilitated RAW264.7 cells to differentiate into osteoclasts in vitro. Moreover, their findings indicated that the facilitation of osteocyte-mediated osteoclastogenesis by HIF-1α via JAK2/STAT3 regulation may be a mechanism for enhancing bone resorption in OA [53]. In addition to hypoxic factors, hormones also have a direct intervention on subchondral bone in the presence of adequate blood supply to subchondral bone through the modulation of JAK2/STAT3 [30]. In a collagenase-induced mouse model of OA, Shao et al. treated the OA model with the intermittent intervention of PTH (1–34), confirming that the inhibition of the JAK2/STAT3 pathway could inhibit subchondral bone remodeling and preserve subchondral bone microarchitecture [31, 32]. Besides, they found that STAT3 inhibitors could suppress RANKL-induced osteoclastogenesis and prevent bone loss caused by ovariectomy, thus mitigating the progression of OA. Consistent with this view, Latourte et al. demonstrated that STAT3 inhibition has chondroprotective effects in the destabilization of the medial meniscus (DMM) mouse model of OA, they observed that STAT3 inhibitors significantly reduced OARSI scores and osteophyte size [54, 55]. In conclusion, the beneficial effects of JAK2/STAT3 inhibition on cartilage include an increase in subchondral bone mass and further protection of the subchondral microarchitecture from deterioration.

### JAK2/STAT3 signaling pathway and synovium in OA

The synovium consists of synovial cells, fibroblasts, and macrophages. Synovitis is a joint lesion in which the synovial membrane is irritated and becomes inflamed, resulting in an imbalance in fluid secretion [56]. In synovitis, the cytokines produced by synovial cells contain interleukin 1 (IL-1), IL-6, interleukin 17 (IL-17), and tumor necrosis factor-α (TNF-α) [57], which increase detrimental mediators and aggravate synovial inflammation and cartilage deterioration [58]. Synovitis is considered a joint lesion in which the synovium is irritated and inflammatory. Hence, it aberrantly affects the pathological progression of OA [59]. JAK2/STAT3 signaling pathway effectively mediates inflammation in OA synovial cells [60]. S100A8/A9 alarmins, recently considered as a joint inflammation marker. Carrion et al. found that IL-22 induced STAT3 phosphorylation and diminished the regulatory effect of IL-22 on S100A8/A9 and MMP1 after inhibiting the activity of JAK2 in fibroblast-like synoviocytes (FLSs) [61]. In another study, Gyurkovska et al. found that JAK2 inhibition reduced inflammation in synovial cells by inhibiting STAT3 phosphorylation [62], and substantially reduced leptin-induced IL-6 and IL-8 production in FLSs [42, 63]. Above all, the JAK2/STAT3 signaling pathway exerts regulatory effects on the synovium in OA. In addition, phosphorylated STAT3, activated by JAK, is translocated to the nucleus, promotes transactivation of receptor activator of nuclear Kappa-B (RANKL) in stromal/osteoblastic cells. CXC-motif ligand (CXCL)16 induced activation of the JAK2/STAT3 pathway, which played a fundamental role in CXCL16-induced RANKL expression. A study compared OA and rheumatoid arthritis (RA) patients and indicated that CXCL16 and RANKL expressions were lower in patients with OA than that in patients with RA. Li et al. [64] showed that CXCL16 was highly expressed in OA synovium and upregulated RANKL expression via the JAK2/STAT3 pathway. The above studies showed that the JAK2/STAT3-mediated regulatory network of chondrocyte, synoviocyte, and osteoblast/osteoclast functions in OA should not be overlooked. It indicates that JAK2/STAT3 plays a pivotal role in the progression of OA and is a potentially vital target in OA treatment.

## Therapeutic approaches to regulate JAK2/STAT3 in OA

**Fig. 4:**
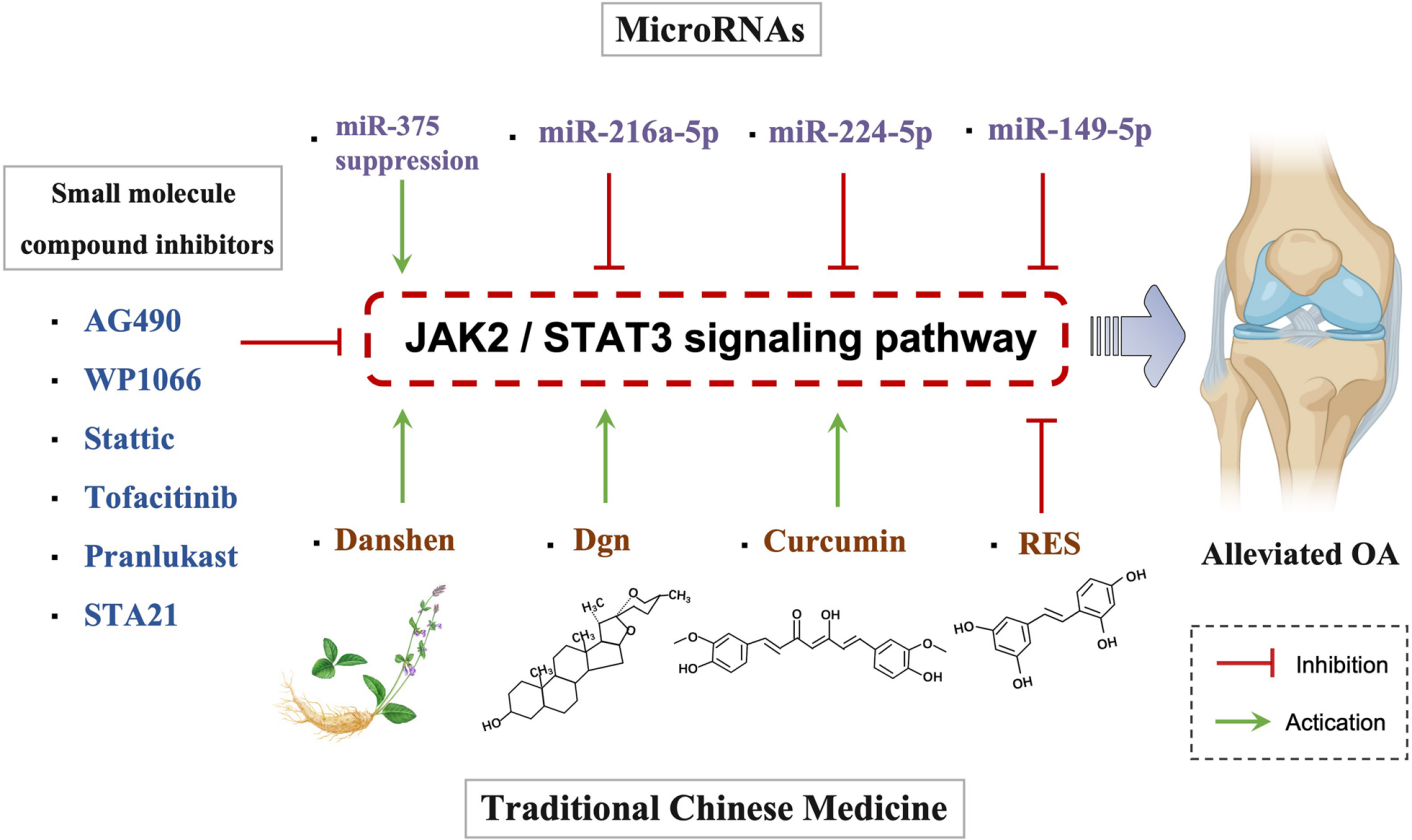
Therapeutic approaches to regulate JAK2/STAT3 signaling pathway in osteoarthritis. MicroRNAs, Traditional Chinese Medicine, and small molecule compound inhibitors are important therapeutic approaches for regulating OA through the JAK2/STAT3 signaling pathway. (Created with BioRender.com.)

Following the understanding of the role of the JAK2/STAT3 pathway in the pathogenesis of OA. Targeting the JAK2/STAT3 pathway for the treatment of OA has been exhibited and illustrated, including microRNAs, traditional Chinese medicine and small molecule compound inhibitors (Fig. 4, Table 2).

**Table 2:** Therapeutic approaches to regulate JAK2/STAT3 in OA

Classification	Name	Effect on JAK2/STAT3	Mechanism	Consequence	References
MicroRNAs	MiR-375 inhibition	activation	ADAMTS-5↓, MMP-13↓, COL-II↑, AGG↑, p-JAK2/JAK2↑, p-STAT3/STAT3↑, Bcl-2/Bax↑	Improved the ability of chondrocytes to antagonize oxidative stress and maintain ECM homeostasis	[28, 67]
	MiR-149-5p	Inhibition	AGT↓, ANGII, AT1R	Regulated and combined AGT, promoted the inflammatory responses induced by IL-6	[39]
	MiR-216a-5p	Inhibition	Bax↓, Caspase-3↓, Bcl-2↑	Promoted the proliferation and migrations well as inhibited apoptosis in chondrocytes	[29]
	MiR-224-5p	Inhibition	MMP-9↓, MMP-13↓, COL-II↑, AGG↑, Caspase-3↓, Circ_PDE1C↓	Inhibited chondrocytes apoptosis and ECM degradation. Targeted CCL2 and accelerated cartilage degradation	[44]
Traditional Chinese medicine	Danshen	activation	P-JAK2, p-STAT3↓, GSH, SOD, CAT↑, Bcl-2↑, Bax, caspase3, PARP↓	Attenuated cartilage injuries by activating JAK2/STAT3 pathway	[71]
	Diosgenin	activation	P-JAK2, p-STAT3, SDH, COX, SOD↑, Bax↓	Protected OA cartilage cells by activating the JAK2/STAT3 pathway, which effectively reduces mitochondrial oxidative stress damage and apoptosis in OA cells during the pathological process	[73]
	curcumin	activation	P-JAK2, p-STAT3,	Increased the mitochondrial resistance to oxidative stress in chondrocytes, significantly slows down the degeneration of articular cartilage, and reduces the level of OA progression	[75]
	Resveratrol	Inhibition	JAK2, STAT3, SOCS3, MMP-13↓	No significant leptin resistance existed in articular cartilage of obesity-related OA and the inhibitory effect of RES on obesity-related OA via alleviating JAK2/STAT3 signaling pathway	[77]
Inhibitors	AG490	Inhibition	JAK2↓	Inhibited JAK2/STAT3 activation and apoptosis in chondrocytes induced by IL-1β	[62, 79]
	WP1066	Inhibition	JAK2, STAT3↓	Downregulated the expression of JAK2/STAT3 to suppress the chondrocytic inflammation responses induced by IL-6	[39]
	Tofacitinibis	Inhibition	JAK, STAT3↓	Moderated chondrogenic hypertrophy in human chondrocyte lines via downregulation of JAK/STAT3	[68]
	Pranlukast	Inhibition	JAK2, STAT1↓	Reversed the enhancement levels of pro-inflammatory cytokines to prevent the progression of OA	[81]
	STA21	Inhibition	STAT3↓	Blocked STAT3 dimerization and DNA binding to alleviate joint pain and cartilage damage	[82]
	Stattic	Inhibition	STAT3↓	Reduced the severity of OA cartilage lesions and decreased the size of osteophytes. Stattic had no significant effect on the synovium or the subchondral bone	[55]

### MicroRNAs

JAK2/STAT3 signaling pathway regulated by microRNAs (miRNAs) can ameliorate the pathogenesis of OA. MiRNAs are endogenous substances that play a gene regulatory role in the process of growth, progression, and disease progression of living organisms, and have become an important new target for new drug progression. An increasing number of studies have highlighted that the microRNAs may serve as a key entry point for the JAK2/STAT3 pathway in the treatment of OA [65, 66]. For example, study has showed that miR-375 inhibitor improved OA chondrocyte metabolism and oxidative stress via activating the JAK2/STAT3 signaling pathway. Zou et al. showed that miR-375 could bind specifically to the 3'UTR region of JAK2 mRNA by dual luciferase reporter analysis, suggesting that JAK2 was a target gene of miR-375. MiR-375 suppression significantly increased the expression of p-JAK2 and p-STAT3 and alleviated damaged chondrocytes, while the protective effect of miR-375 suppression was reversed after JAK2 siRNA treatment. Thus, the miR-375 inhibitor could protect chondrocytes, antagonize oxidative stress, suppress apoptosis, and maintain ECM homeostasis by activating the JAK2/STAT3 signaling pathway [67]. Similarly, Lu et al. has found that the epigenetic silencing of miR-375 involved cartilage degradation by targeting the JAK2/STAT3 pathway in OA [28]. Besides, Wang et al. reported that miR-149-5p, a downregulated miRNA in OA cartilage tissue, also decreased IL-6-induced chondrocyte inflammation in OA. They found that Angiotensinogen (AGT) was targeted by miR-149-5p, which promoted IL-6-induced inflammatory responses in osteoarthritic chondrocytes by activating the JAK2/STAT3 pathway. Therefore, they concluded that under the regulation and immediate association of miR-149-5p, AGT facilitated IL-6-induced inflammatory response in OA through JAK2/STAT3 pathway. In another vivo experiment, Chiu et al. found that the high expression of miR-149-5p c restore cartilage homeostasis and thus arrest cartilage hypertrophy in human chondrocyte cell lines C20A4 and C28/I2 [68]. Another miRNA, miR-216a-5p, also played an important role through the JAK2/STAT3 signaling pathway in OA. The role of miR-216a-5p in SEV acquired from bone marrow mesenchymal stem cells under hypoxia and their potential biological mechanisms to promote osteoarthritis repair in vivo has attracted attention. Rong et al. showed that JAK2 was identified as the target gene of miR-216a-5p, and suppression of JAK2 enhanced the inhibitory effect of miR-216a-5p-Hypo-sEV on IL-1β-triggered chondrocytes apoptosis. Hypo-sEV miR-216a-5p could promote chondrocyte proliferation, and migration, and reduce apoptosis via inhibiting the JAK2/STAT3 signaling pathway [29]. Moreover, the role of miRNA and lncRNA interactions in the JAK2/STAT3 pathway is also gradually being elucidated. For example, Inc RNA DANCR mediated the pathological process of OA chondrocytes through acting as a competitive endogenous RNA for miR-216a-5p. Inc RNA DANCR inhibition suppressed proliferation, and inflammation, and promoted apoptosis of OA chondrocytes through the miR-216a-5p/JAK2/STAT3 signaling pathway. Additionally, circRNAs are engaged in pathogenesis by robust binding and repression of miRNA transcription, which subsequently impacted downstream mRNA expression. Yao et al. found that the knockdown of circ-PED1C could promote the mitigation of cartilage degeneration via sponging miR-224-5p [45]. While CCL2 was targeted by miR-224-5p, which activated the JAK2/STAT3 signaling pathway, thus leading to cartilage degradation and exacerbating the pathological process of OA [44]. Given the promising future of miRNAs in OA therapy and drug progression through the JAK2/STAT3 pathway, research and progression work is in full swing, and revealing their mechanism of action in diseases is a key and difficult issue that needs to be investigated in depth in order to fully develop their role in disease therapy.

### Traditional Chinese medicine

A growing number of studies show that Traditional Chinese medicine (TCM) has been accepted as an effective complementary therapy for OA and is expected to be the next generation of therapeutic drugs as an alternative to synthetic compounds, which can alleviate the symptom of OA through the JAK2/STAT3 pathway [69]. TCM is mainly derived from natural medicine and its extracts, with the theory of Chinese traditional medicine guiding the clinical application of the drug [2]. Before the invention of extractive and synthetic chemistry, musculoskeletal disorders were treated with TCM [70]. For instance, Danshen is one of the TCM, which can suppress apoptosis of primary chondrocytes and promote chondrocyte proliferation. In animal experiments, Xu et al. found that Danshen (1.05 g/day) or sodium hyaluronate (SH) significantly elevated the expression of p-JAK2 and p-STAT3 in the OA rabbits. This implied that the role of Danshen was related to the JAK2/STAT3 signaling pathway. At the same time, they established an in vitro OA model by exposing chondrocytes isolated from normal rabbit cartilage to sodium nitroprusside (SNP). The phosphorylation levels of JAK2 and STAT3 were reduced after SNP treatment, which was able to be rescued by Danshen. Furthermore, inhibitors of JAK2 antagonized the anti-apoptotic effects of Danshen, which further suggested that Danshen could restore OA cartilage degeneration by activating the JAK2/STAT3 signaling pathway. However, it is unclear which component of Danshen plays a key role in this process [71]. Although the main components in Danshen affecting OA have not been proven, certain components of TCM have now been shown to be beneficial in delaying the pathological process of OA. For example, Diosgenin (Dgn) is a steroidal saponin abundant in Dioscorea opposite, which has the function of promoting the proliferation and differentiation of osteoblasts and inhibiting the formation of osteoclasts [72]. A previous study has proved that Dgn exerted anti-OA effects by inhibiting the expression of NF-kB and oxidative stress induced by human monocyte line THP-1 cells, but its mechanism of action had not been fully elucidated, while NF-kB and JAK2/STAT3 signaling pathway were associated [35]. Recently, the relationship between Dgn and JAK2/STAT3 signaling pathway in OA has also been reported. Liu et al. administered the Dgn intraperitoneally (100 mg/kg/day) to the monosodium iodoacetate (MIA)-induced OA mice model, they found that Dgn significantly increased the protein expression levels of p-JAK2 and p-STAT3, effectively reducing chondrocyte damage during OA pathology. The inhibition of JAK2/STAT3 could reverse the protective effect of Dgn. Thus, Dgn exhibited protective effects against cartilage destruction via activating the JAK2/STAT3 pathway [73]. Besides, curcumin is a compound extracted from turmeric, a yellow pigment known for its medicinal properties. Curcumin not only activated monocytes and macrophages and released lysozyme by inhibiting MCP-1, but also upregulated monocyte and macrophage adhesion molecules and IL-1 and IL-6 levels [74]. Li et al. constructed a mouse model of OA and administered curcumin (100 mg/kg) intraperitoneally to OA mice daily for 4 weeks. They found that curcumin could effectively promote the activation of the JAK2/STAT3 signaling pathway, inhibit chondrocyte apoptosis, and improve mitochondrial resistance to oxidative stress in chondrocytes [75]. The above studies confirmed that TCM components played a positive role in improving OA through the JAK2/STAT3 pathway, whereas TCM compounds are complex and contradictory reports appeared. For example, Resveratrol (RES) has been reported to alleviate OA by inhibiting the JAK2/STAT3 pathway. RES is a natural phenolic compound, which has protective effects against osteoarthritis, including anti-apoptotic, anti-inflammatory, and antioxidant effects [76]. Jiang et al. found that RES (45 mg/kg) prevented the progression of obesity-related OA in high fat diet models in C57BL/6 J mice. This progression was achieved by inhibition of the JAK2/STAT3 pathway by RES in cartilage. In in vitro study, RES exhibited the same effect in leptin-stimulated human osteosarcoma cells SW1353 cells by blocking the expression of p-JAK2 and p-STAT3. This suggested that RES acts by inhibiting the JAK2/STAT3 signaling pathway, and the specific mechanism of RES needed to be further investigated [77]. All of the above studies indicated that TCM alleviates OA through JAK2/STAT3 signaling pathway, implying that TCM may represent a promising strategy for the treatment of OA progression by regulating the JAK2/STAT3 signaling pathway. More TCM components acting on the JAK2/STAT3 signaling pathway have yet to be identified, and the mechanism of action of TCM components regulating OA through the JAK2/STAT3 signaling pathway needs to be further investigated.

### Small molecule compound inhibitors

Apart from microRNAs, various small molecule compound inhibitors of JAK2/STAT3 appear to be efficacious in OA and hold promise as future therapeutic options, particularly in the progression of potential new drugs and effective therapeutics. For example, AG490 acts as an inhibitor of JAK2 to improve cartilage and bone damage. This effect is associated with reduced expression of STAT3 and phosphorylated JAK2 in the joints of arthritic mice [62]. Yao et al. [78] showed that AG490 (100 nM) significantly inhibited IL-1β-induced expression of JAK2 and STAT3 in primary chondrocytes of SD rats. Other studies showed that AG490 has a beneficial action against OA, Zhang et al. found that apoptosis and autophagy-related protein expression can be altered by AG490 after leptin-induced chondrocyte damage [79, 80]. Liu et al. [30] showed that AG490 (10 µm) could downregulate p-JAK2 and p-STAT3 to improve the damaged articular cartilage matrix. In addition to AG490, other inhibitors of JAK2/STAT3 had a reversal effect on cartilage degeneration, for instance, WP1066 was identified as a JAK2/STAT3 inhibitor, which rescued the IL-6-induced phosphorylation of JAK2 and STAT3 levels. Wang et al. showed that WP1066 could downregulate the expression of JAK2/STAT3 to suppress the chondrocyte inflammation responses induced by IL-6 [39]. Analogously, Chiu et al. showed that tofacitinib is one of the inhibitors targeting JAK and STAT3. The SD rats received intra-articular injection of tofacitinib (10 mg/kg, once per week for 4 weeks), which could upregulate the expression of miR-149-5p and moderate chondrogenic hypertrophy by downregulation of JAK/STAT3 [68]. Notably, there is a type of small molecule inhibitor, Pranlukast (10 µm), which could suppress the activation of the JAK2, so that reversing the enhancement levels of pro-inflammatory cytokines to prevent the progression of OA [81]. Recently, small molecule inhibitors that specifically inhibit STAT3 have also emerged. In an MIA-induced OA model, Lee et al. found that STA21, an inhibitor of STAT3 blocked STAT3 dimerization and DNA binding to alleviate joint pain and cartilage damage [82]. Another small molecule inhibitor of STAT3, Stattic, significantly represses its activation and nuclear translocation. Stattic has a similar role in restraining STAT3 phosphorylation and as a promising generation of osteoclast inhibitors. Li et al. showed that Stattic inhibited RANKL-mediated osteoclastogenesis and bone loss in vitro and vivo studies. However, Stattic had no significant effect on the synovium or subchondral bone [54, 55]. Overall, these findings demonstrated that diverse JAK2/STAT3 inhibitors have their own correspondingly different roles, further indicating that OA could be healed by directly or indirectly targeting JAK2/STAT3, but the clinical study is still needed.

## Conclusion and perspectives

As described in this review, JAK2/STAT3 is a complicated signaling pathway with several regulators and effectors. The JAK2/STAT3 signaling pathway plays an instrumental role in the osteoarticular system, including cartilage, subchondral bone, and synovium. Most significantly, this signaling is significant for OA progression.

According to existing studies, targeting the JAK2/STAT3 pathway is a feasible treatment for OA. However, there are still gaps in the specific regulatory role of JAK2/STAT3 in OA. Although most studies have shown that inhibition of the JAK2/STAT3 signaling pathway alleviates OA pathology, still some studies have reported that activation of the JAK2/STAT3 signaling pathway facilitates recovery from OA, such as inhibition of miR-375, Danshen, Dgn and curcumin. Simply activating or inhibiting JAK2/STAT3 to counteract OA may be a double-edged sword, as different regulatory factors can alleviate OA by activating or inhibiting the JAK2/STAT3 pathway. For example, some herbs activate JAK2/STAT3, while some hormones inhibit the pathway to arrest the progression of OA, where the side effects produced by JAK2/STAT3 are not known. Moreover, most of the positive side effects of the JAK2/STAT3 signaling pathway come from different tissues in mammals. In other words, the current experimental design is mostly limited to preclinical studies such as cellular and animal experiments, while the clinical application of JAK2/STAT3 signaling in patients is still scarce. Accurate and efficient drug delivery is also a challenge that cannot be ignored. Therefore, it is imperative to elucidate the functions and more specific molecular mechanisms of the JAK2/STAT3 signaling pathway in different pathophysiological stages of OA. In recent years, physical factor therapy has become a clinical hot therapy for bone-related diseases. A novel idea is that JAK2/STAT3 signaling can be combined with physical factor therapy to improve OA, including Pulsed electromagnetic field (PEMF), extracorporeal shock wave treatment (ESWT), and engineered cartilage and regenerative techniques, further developing more effective applications for the treatment of OA. The JAK2/STAT3-based therapies for OA become safe and valid when these intractable points are addressed.

Therefore, the combined efforts of more relevant scholars and experts are needed to conduct experimental analyses and to develop an operative structure so that JAK2/STAT3 signaling pathway can more effectively benefit patients with OA. It is hoped that increasingly extensive and well-designed pharmacological and clinical studies will be based on conclusive evidence for multiple effective and comprehensive targeting of the JAK2/STAT3 signaling pathway.

## Data Availability

All data generated or analysed during this study are included in this published article.

## Abbreviations

OA: Knee osteoarthritis
JAK2: Janus kinase 2
STAT3: Signal transduction and activator of transcription 3
ECM: Extracellular matrix
Bcl-2: B-cell lymphoma-2
Bax: Bcl-2 Associated X protein regulator
caspase-3: Cysteine aspartate protease-3
caspase-9: Cysteine aspartate protease-9
iNOS: Nitric oxide synthase
ROS: Reactive oxygen species
Bcl-1: B-cell lymphoma-1
LC3-II: Microtubule-associated protein light chain 3
COL-II: Type II collagen
AGG: Aggregate
IL-β: Interleukins-β
IL-6: Interleukins-6
IL-7: Interleukins-7
IL-8: Interleukins-8
IL-17A: Interleukins-17A
Bax: Bcl-2 Associated X protein regulator
GSH: Glutathione
SOD: Superoxide dismutase
CAT: Catalase
MMP-13: Matrix metalloproteinase-13
ADAMTs-14/15: A disintegrin and metalloproteinase with thrombospondin motifs-14/15
COL-II: Type II collagen
AGG: Aggregate
TNF-α: Tumor necrosis factor-α
ACLT: Anterior cruciate ligament transection
iNOS: Nitric oxide synthase
COX-2: Cyclo-oxygenase2
DANCR: Differentiation antagonizing non-protein coding RNA
KLF4: Kruppel-like factor (KLF)-4
INSR: Insulin receptor
sEVs: Small extracellular vesicles
TLR4: Toll-like receptor 4
FLS: Fibroblast-like synoviocyte
PTH(1–34): Parathyroid hormone(1–34)
TRIM59: The tripartite motif 59
INSR: Insulin receptor
DUSP19: Dual specificity protein 19
DA: Dopamine
AG490: A specific inhibitor of JAK2
WP1066: An inhibitor of JAK2/STAT3 and a derivative of AG490
MIA: Sodium iodoacetate
Ghrelin: A 28 amino-acid secreted peptide hormone
HIF‐1α: Hypoxia‐inducible factor‐1α
Stattic: A small-molecule specific inhibitor of STAT3 IL-22 belongs to the IL-10 superfamily of cytokines and signals and mainly activating the STAT3
CCL2: C–C motif chemokine ligand 2
CXCL16: CXC-motif ligand 16
FLSs: Fibroblast-like synoviocytes
RES: Resveratrol
Dgn: Diosgenin
IRS-1: Insulin-receptor substrate 1
PI3K: Phosphatidylinositol-3-kinase
Akt: Protein kinase B
MAPK: Mitogen-activated protein kinase
NF-kB: Nuclear factor-kappa B
Dgn: Diosgenin
SNP: Sodium nitroprusside
SH: Sodium hyaluronate
RES: Resveratrol
